# Outpatient shoulder arthroplasty: An updated systematic review, meta-analysis, and trial sequential analysis on clinical outcomes and cost-effectiveness

**DOI:** 10.1177/17585732251349754

**Published:** 2025-06-25

**Authors:** Ahmed Al-Saadawi, Sam Tehranchi, Richard Benson, David Rose, Obinna Jude Nzeako

**Affiliations:** 1 School of Medicine, Faculty of Medicine and Dentistry, 4952 Queen Mary University of London, London, England; 2 Consultant Trauma & Orthopaedic Surgeon, Department of Trauma and Orthopaedic Surgery, Maidstone Hospital, 6484 Maidstone and Tunbridge Wells NHS Trust, London, England

**Keywords:** shoulder arthroplasty, outpatient, ambulatory, same-day discharge

## Abstract

**Background:**

Outpatient shoulder arthroplasty has become increasingly popular in recent years. The aim of this study was to compare clinical outcomes and procedural costs between outpatient and inpatient shoulder arthroplasty.

**Methods:**

Five databases were searched from their inception to November 7^th^, 2024. Meta-analysis and trial sequential analysis were conducted to compare complications, readmission, revision surgery, and emergency department attendance between the two approaches. Procedural costs were reported descriptively.

**Results:**

Thirty-four studies were included in the review. The meta-analysis revealed that medical complications (OR: 0.59, P = 0.0004) were significantly lower in the outpatient setting. No significant differences in total complications (OR: 0.74, P = 0.06), surgical complications (OR: 0.90, P = 0.59), readmission (OR: 1.08, P = 0.67), revision surgery (OR: 0.85, P = 0.15), or emergency department attendance (OR: 0.93, P = 0.68) were observed between the two approaches. The trial sequential analysis indicated that only the meta-analysis of total complications met the required information size to be considered conclusive and not at risk of random error. Outpatient surgery was associated with significantly lower procedural costs.

**Conclusion:**

Outpatient shoulder arthroplasty is cost-effective and can yield non-inferior outcomes compared to the inpatient approach. However, further research is required to strengthen the evidence base.

**Level of evidence:**

IV

## Introduction

In recent years, shoulder arthroplasty (SA) has seen a marked growth in popularity, with over 8000 procedures performed annually in the United Kingdom (UK) and more than 100,000 in the United States of America (USA). This figure is projected to dramatically increase in coming years.^1–3^ It is utilised in the management of a broad range of glenohumeral pathologies.^4^ Total shoulder arthroplasty (TSA), divided into anatomic (aTSA) and reverse (rTSA) techniques, accounts for most procedures and significantly improves pain, range of motion, and subsequent quality of life in affected patients.^5^ With advancements in surgical techniques and implant design, SA has become increasingly safe, with low rates of peri-operative complications and shortened inpatient stays.^6^ However, particularly in implant design, these advancements have been accompanied by a significant increase in costs.^7^ The anticipated rise in procedural volume over the next decade, coupled with rising costs, is likely to heighten pressure on healthcare institutions globally, many of which are already overburdened.^2,8^

In response to these challenges, there has been a transition towards performing SA in the outpatient setting.^9^ The need for such change was amplified by the COVID-19 (Coronavirus Disease 2019) pandemic, which caused a major backlog in elective procedures. On January 1^st^, 2021, TSA was removed from the inpatient-only list for Centers for Medicare and Medicaid Services to address such unprecedented circumstances.^10^ Underscoring the pandemic's role in driving this paradigm shift, Seetharam et al. reported an increase in outpatient SA from 4.5% in the pre-COVID era to 31.8% in the post-COVID era (defined as March 2020 and later).^11^

Prior systematic reviews and meta-analyses have already demonstrated comparable clinical outcomes and cost-effectiveness with outpatient SA; however, their last search dates were limited to 2020.^12–14^ With the volume of outpatient procedures increasing substantially since then, and multiple newly published studies,^11,15,16^ an updated systematic review and meta-analysis is warranted. This study aimed to evaluate and compare post-operative complications, readmissions, revision surgery rates, emergency department (ED) attendances, and procedural costs between outpatient and inpatient SA. Additionally, a trial sequential analysis was performed on each meta-analysis to control for Type I and II errors and determine whether the effect was large enough to be considered conclusive.^17^

## Methods

### Protocol and registration

This review adhered to the preferred reporting items for systematic reviews and meta-analyses (PRISMA) guidelines.^18^ The protocol was submitted to PROSPERO (International Prospective Register of Systematic Reviews) on November 6^th^, 2024 (PROSPERO ID: CRD42024608936).

### Eligibility criteria

#### Inclusion

*Study Design*: Randomised controlled trials, case controls, case series, prospective, and retrospective cohort studies.

*Population*: Patients aged 18 years or older who required shoulder arthroplasty: defined as either aTSA, rTSA, or shoulder hemiarthroplasty.

*Intervention*: Shoulder arthroplasty was performed as an outpatient procedure. To meet the criteria for an *outpatient procedure*, patients must have been discharged home after a length of stay of less than 24 h.

*Comparator*: Studies must include a comparator group of patients undergoing shoulder arthroplasty in an inpatient setting.

*Outcome*: The assessed outcomes included total post-operative complications, surgical and medical complications, 90-day readmissions, 90-day revision surgery rates, 90-day ED attendances, and procedural costs.

#### Exclusion

- Case reports, editorials, abstracts, commentaries, expert opinions, and review articles.
- Studies involving animal or cadaveric participants.
- Studies that did not include a comparator group of patients undergoing inpatient shoulder arthroplasty.
- Studies that did not report the aforementioned outcomes.
- Studies written in languages other than English.

### Information sources and search strategy

Five databases were comprehensively searched from their inception to November 7^th^, 2024: MEDLINE, Scopus, Embase, Web of Science, and CENTRAL. The primary terms employed in our search strategy were “outpatient”, “shoulder”, and “arthroplasty”. The remainder of this strategy can be found in the supplementary files.

### Study selection

Titles and abstracts were imported into a reference manager, where duplicate articles were identified and excluded. The remaining articles were then uploaded onto Rayyan,^19^ where further duplicates were removed. Two reviewers (AS and ST) independently screened the articles on Rayyan, tagging each article as *include*, *maybe*, or *exclude*. Articles tagged as *include* by both authors advanced to full-text review, whereas articles tagged as *include* by only one author or tagged as *maybe* by both authors were resolved through discussions. The remaining articles were excluded from the analysis. A third reviewer (ON) made the final decision if any disagreements could not be resolved between the two primary reviewers.

### Data extraction

Two independent reviewers (AS and ST) extracted data from the included articles using a bespoke form. The extracted variables included: (1) authors, (2) study details and design, (3) data source, (4) sample size, (5) patient demographics, (6) arthroplasty technique, (7) post-operative complications, (8) readmissions, (9) revision surgeries, (10) ED attendance, and (11) procedural costs.

### Risk of bias

The risk of bias was assessed using the Risk of Bias in Non-randomised Studies – of Interventions (ROBINS–I) tool. It consists of a three-stage assessment process: pre-intervention, at-intervention, and post-intervention. Amongst them, there are seven distinct domains assessed. The risk of bias in each domain was rated using five response options: *low* risk, *moderate* risk, *serious* risk, *critical* risk, or *no information*. An overall judgement of the risk of bias was determined based on the ratings of individual domains, using the same set of response options.^20^

### Statistical analysis

Patient demographics, including age, sex, diabetes, American Society of Anaesthesiologists (ASA) classification, body mass index (BMI), and smoking status, were pooled to provide overall estimates. Meta-analyses were conducted using a DerSimonian-Laird random-effects approach on the RevMan 5.4 software. Post-operative complications — including total, surgical and medical complications — along with readmission, revision surgery, and ED attendance were all reported as dichotomous outcomes and expressed as an odds ratio (OR) with 95% confidence intervals (CI). A pre-planned subgroup analysis was conducted for each meta-analysis to classify studies based on their sources of patient data: institutional records or databases. Additionally, a post-hoc sensitivity analysis was performed to examine post-operative complications specifically in studies reporting same-day discharge. Heterogeneity was assessed using *Cochran's Q Test*, where an I^2^ score less than 25% suggests low heterogeneity, 25%–50% represents moderate heterogeneity, and greater than 50% indicates high heterogeneity.

Trial sequential analysis was conducted using the Trial Sequential Analysis software (Copenhagen Trial Unit, Centre for Clinical Intervention Research, Copenhagen) on six outcomes: post-operative complications, surgical complications, medical complications, readmission, revision surgery, and ED attendance. Given the overlap in database sources in the existing literature on outpatient SA, a decision was made to include only studies using data from their institutions in the TSA to ensure accuracy in data reporting. The required information size (RIS) for each outcome was estimated based on the effect size in the corresponding meta-analysis, using a Type I error threshold of 5% and a statistical power of 90%. The O’Brien-Fleming α-spending function was used to draw monitoring boundaries, including the futility boundaries (inner wedge).

## Results

**Figure 1.**
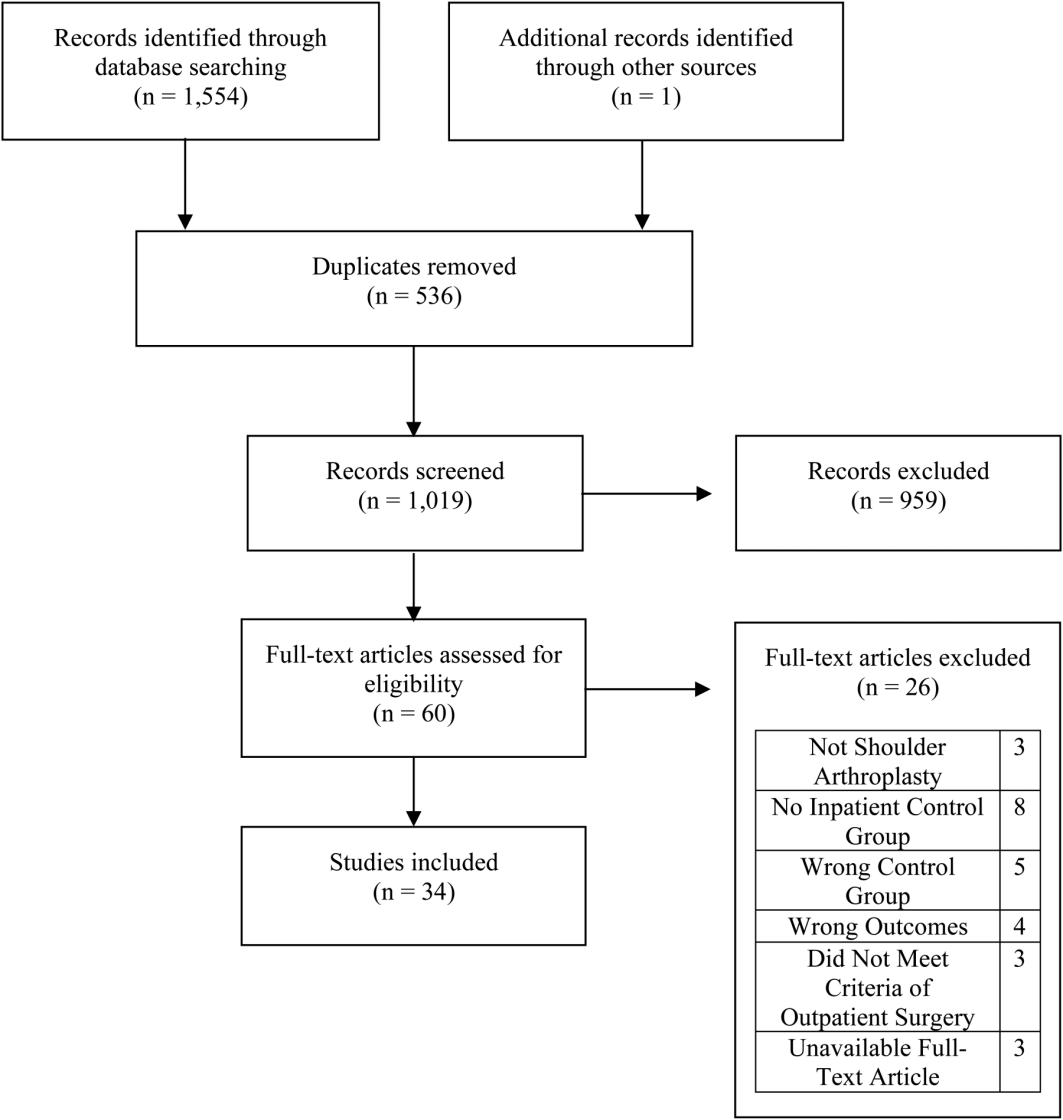
PRISMA flow chart illustrating selection of studies.

Our literature search identified 1555 articles, which was reduced to 1019 after removing duplicates. Following the initial screening phase, 60 articles were deemed eligible for full-text review. Of these, 3 were excluded because they did not focus solely on shoulder arthroplasty, 8 lacked a comparator inpatient group, and 4 did not report the desired outcomes. Full-text articles were unavailable for 3 studies and were therefore also excluded. Thirty-four studies met the inclusion criteria and were included in the final analysis (Figure 1).

### Characteristics of included studies

The 34 included studies^11,15,16,21–51^ encompassed 428,127 patients, of whom 36,711 underwent shoulder arthroplasty in an outpatient setting, while 391,416 were managed as inpatients. All studies were considered to have a *serious* risk of bias, except for O’Donnell et al.^50^ (Online Appendix 1; Online Appendix A). Most studies took place in the USA, with the exception of six that took place in the UK (2), Canada, France, Australia, and the Netherlands respectively. Flurin et al.,^16^ van Kampen et al.,^48^ and Gallay et al.^51^ employed a prospective cohort design, while the remaining utilised a retrospective cohort. Fifteen studies collected patient data from national databases, whereas 19 used their institutional records. The American College of Surgeons National Surgical Quality Improvement Program (ACS-NSQIP) was the most frequently used database amongst the included studies. Twenty-three studies examined patients who underwent either aTSA or rTSA, six focused exclusively on aTSA, and two on rTSA. Three studies broadened their inclusion criteria to incorporate shoulder hemiarthroplasties in addition to aTSA and rTSA. Table 1 provides a detailed summary of the studies included.

**Table 1.** Summary of included studies.

Study / Setting	Study Design	Sample Size	Data Characteristics	Procedure	Outcomes	Follow-Up	ROBINS-I
Antonacci et al., 2020, USA^21^	Retrospective cohort	**Outpatient:** 52 **Inpatient:** 92	**Source of Data:** Institutional **Duration**: 2016 – 2019	rTSA	**•** Complications **•** Readmission	90 Days	Serious risk
Agarwal et al., 2023, USA^22^	Retrospective cohort	**Outpatient:** 1613 **Inpatient:** 3218	**Source of Data**: PearlDiver Patient Record Database **Duration**: 2010 – 2021	aTSA + rTSA	**•** Complications **•** Readmission **•** ED attendance **•** Procedural costs	90 Days, 5 Years	Serious risk
Arshi et al., 2018, USA^23^	Retrospective cohort	**Outpatient**: 1555 **Inpatient**: 15,987	**Source of Data**: PearlDiver Patient Record Database **Duration**: 2007 – 2016	aTSA + rTSA	**•** Complications	1 Year	Serious risk
Basques et al., 2017, USA^24^	Retrospective cohort	**Outpatient**: 3493 **Inpatient**: 119,854	**Source of Data**: United States Medicare Standard Analytical File **Duration**: 2005 – 2012	aTSA + rTSA	**•** Complications **•** Readmission	90 Days	Serious risk
Bean et al., 2018, USA^25^	Retrospective cohort	**Outpatient**: 21 **Inpatient**: 40	**Source of Data**: Institutional **Duration**: N/A	aTSA + rTSA	**•** Complications **•** Readmission **•** ED attendance **•** Revision surgery	90 Days	Serious risk
Borakati et al., 2020, United Kingdom^26^	Retrospective cohort	**Outpatient:** 18 **Inpatient:** 41	**Source of Data:** Institutional **Duration**: 2017 – 2018	aTSA + rTSA	**•** Complications **•** Readmission **•** ED attendance **•** Procedural costs	30 Days, 90 Days	Serious risk
Brolin et al., 2016, USA^27^	Retrospective cohort	**Outpatient**: 30 **Inpatient**: 30	**Source of Data**: Institutional **Duration**: 2012 – 2015	aTSA	**•** Complications **•** Readmission **•** Revision surgery	90 Days	Serious risk
Cancienne et al., 2017, USA^28^	Retrospective cohort	**Outpatient:** 706 **Inpatient:** 4459	**Source of Data**: PearlDiver Patient Record Database **Duration**: 2010 – 2014	aTSA	**•** Complications **•** Readmission **•** Procedural costs	90 Days	Serious risk
Cronin et al., 2022, USA^29^	Retrospective case series	**Outpatient:** 38 **Inpatient:** 989	**Source of Data**: Institutional **Duration**: 2015 – 2020	aTSA + rTSA	**•** Procedural costs	N/A	Serious risk
Carbone et al., 2021, USA^30^	Retrospective cohort	**Outpatient:** 3947 **Inpatient:** 104,942	**Source of Data**: Centers for Medicare & Medicaid Services Limited Data Set files **Duration**: 2016 – 2018	aTSA + rTSA	**•** Readmission **•** Procedural costs	90 Days	Serious risk
Elgalli et al., 2022, United Kingdom^31^	Retrospective cohort	**Outpatient:** 46 **Inpatient:** 36	**Source of Data:** Institutional **Duration**:2014 – 2019	aTSA + rTSA + HSA	**•** Complications **•** Revision surgery	180 Days, Annually	Serious risk
Erickson et al., 2020, USA^32^	Retrospective cohort	**Outpatient:** 94 **Inpatient:** 77	**Source of Data:** Institutional **Duration**: 2015 – 2017	aTSA	**•** Complications **•** Readmission **•** Revision surgery	Minimum 2 Years	Serious risk
Erickson et al., 2020, USA^33^	Retrospective cohort	**Outpatient**: 241 **Inpatient**: 373	**Source of Data**: Institutional **Duration**: 2015 – 2017	rTSA	**•** Complications **•** Readmission **•** Revision surgery	Minimum 2 Years	Serious risk
Flurin et al., 2024, France^16^	Prospective cohort	**Outpatient:** 106 **Inpatient:** 59	**Source of Data**: Institutional **Duration**: 2019 – 2021	aTSA + rTSA	**•** Complications **•** Readmission **•** Revision surgery	90 Days	Serious risk
Gregory et al., 2019, USA^34^	Retrospective cohort	**Outpatient:** 1542 **Inpatient:** 21,331	**Source of Data**: Texas Health Care Information Collection database **Duration**: 2010 – 2015	aTSA + rTSA	**•** Procedural costs	N/A	Serious risk
Gallay et al., 2008, Canada^51^	Prospective cohort	**Outpatient:** 8 **Inpatient:** 8	**Source of Data:** Institutional **Duration**: 2004 – 2006	aTSA	**•** Readmission **•** ED attendance	Up to 32 months	Serious risk
Guareschi et al., 2023, USA^35^	Retrospective cohort	**Outpatient:** 180 **Inpatient:** 1276	**Source of Data**: American College of Surgeons National Surgical Quality Improvement Program database **Duration**: 2010 – 2019	aTSA + rTSA	**•** Complications **•** Readmission **•** Revision surgery	30 Days	Serious risk
Hachadorian et al., 2023, USA^36^	Retrospective cohort	**Outpatient:** 1005 **Inpatient:** 809	**Source of Data**: Kaiser Permanente's Shoulder Arthroplasty Registry (SAR) **Duration**: 2018 – 2020	aTSA + rTSA	**•** Complications **•** Readmission **•** ED attendance	90 Days	Serious risk
Harris et al., 2021, USA^37^	Retrospective cohort	**Outpatient:** 1714 **Inpatient:** 1714	**Source of Data**: American College of Surgeons National Surgical Quality Improvement Program database **Duration**: 2010 – 2017	aTSA + rTSA	**•** Readmission	30 Days	Serious risk
Jennewine et al., 2024, USA^38^	Retrospective cohort	**Outpatient:** 108 **Inpatient:** 671	**Source of Data**: Institutional **Duration**: 2009 – 2023	aTSA + rTSA	**•** Complications **•** Readmission **•** Revision surgery	Minimum 2 Years	Serious risk
Kramer et al., 2020, USA^39^	Retrospective cohort	**Outpatient**: 405 **Inpatient**: 6098	**Source of Data**: Kaiser Permanente's Shoulder Arthroplasty Registry (SAR) **Duration**: 2005 – 2016	aTSA + rTSA	**•** Complications **•** Readmission **•** ED attendance	90 Days	Serious risk
Kucharik et al., 2022, USA^40^	Retrospective cohort	**Outpatient:** 1328 **Inpatient:** 18,707	**Source of Data**: American College of Surgeons National Surgical Quality Improvement Program database **Duration**: 2007 – 2019	aTSA + rTSA	**•** Complications **•** Readmission	30 Days	Serious risk
Leroux et al., 2016, USA^41^	Retrospective cohort	**Outpatient**: 173 **Inpatient**: 7024	**Source of Data**: American College of Surgeons National Surgical Quality Improvement Program database **Duration**: 2005 – 2014	aTSA + rTSA	**•** Complications **•** Readmission	30 Days	Serious risk
Nwankwo et al., 2018, USA^42^	Retrospective cohort	**Outpatient**: 118 **Inpatient**: 64	**Source of Data**: Institutional **Duration**: 2012 – 2016	aTSA + rTSA	**•** Readmission **•** ED attendance	90 Days	Serious risk
Nelson et al., 2019, USA^43^	Retrospective cohort	**Outpatient:** 35 **Inpatient:** 46	**Source of Data**: Institutional **Duration**: 2012 – 2015	aTSA	**•** Complications **•** Readmission **•** ED attendance	90 Days	Serious risk
Ode et al. 2020, USA^44^	Retrospective cohort	**Outpatient:** 974 **Inpatient**: 37,881	**Source of Data**: **•** State Inpatient Databases **•** State Ambulatory Surgery Databases **Duration**: 2010 – 2014	aTSA + rTSA	**•** Readmission **•** Total procedural costs	90 Days	Serious risk
O’Donnell et al., 2024, USA^50^	Retrospective cohort	**Outpatient:** 14,540 **Inpatient:** 40,576	**Source of Data**: Centers for Medicare & Medicaid Services Limited Data Set files **Duration**: 2019 – 2022	aTSA + rTSA	**•** Complications **•** Readmission	90 Days	Moderate risk
Posner et al., 2023, USA^45^	Retrospective cohort	**Outpatient:** 94 **Inpatient:** 24	**Source of Data**: Institutional **Duration**: 2017 – 2022	aTSA	**•** Complications **•** Readmission	90 Days	Serious risk
Raji et al., 2024, USA^46^	Retrospective cohort	**Outpatient:** 174 **Inpatient:** 121	**Source of Data**: Institutional **Duration**: 2018 – 2021	aTSA + rTSA	**•** Complications **•** Readmission **•** Revision surgery **•** ED attendance **•** Total costs	90 Days	Serious risk
Rizvi et al., 2023, Australia^47^	Retrospective case-controlled	**Outpatient**: 37 **Inpatient**: 36	**Source of Data:** Institutional **Duration**: 2004 – 2012	aTSA + rTSA + HSA	**•** Complications **•** Readmission	Minimum 2 Years	Serious risk
Seetharam et al., 2022, USA^11^	Retrospective cohort	**Outpatient:** 86 **Inpatient:** 184	**Source of Data**: Institutional **Duration**: 2018 – 2021	aTSA + rTSA	**•** Complications **•** Readmission **•** ED attendance	90 Days	Serious risk
Trudeau et al., 2022, USA^15^	Retrospective cohort	**Outpatient:** 2185 **Inpatient:** 20,357	**Source of Data**: American College of Surgeons National Surgical Quality Improvement Program database **Duration**: 2015 – 2019	aTSA + rTSA	**•** Complications **•** Readmission **•** Revision surgery	30 Days	Serious risk
van Kampen et al., 2022, Netherlands^48^	Prospective cohort	**Outpatient:** 51 **Inpatient:** 112	**Source of Data**: Institutional **Duration**: 2018 – 2021	aTSA + rTSA + HSA	**•** Complications **•** Readmission **•** ED attendance	90 Days	Serious risk
Willenbring et al., 2021, USA^49^	Retrospective cohort	**Outpatient:** 47 **Inpatient:** 98	**Source of Data**: Institutional **Duration**: 2015 – 2020	aTSA + rTSA	**•** Complications **•** Readmission **•** ED attendance	90 Days	Serious risk

**Legend:** aTSA: Anatomic total shoulder arthroplasty; rTSA: Reverse total shoulder arthroplasty; HSA: Hemiarthroplasty

### Patient demographics

Patient demographics were reported in all studies. Agarwal et al.,^22^ Brolin et al.,^27^ Cancienne et al.,^28^ and Harris et al.^37^ matched the inpatient and outpatient cohort characteristics. The remaining studies reported unmatched demographics of each cohort, which are pooled and summarised in Online Appendix 2 (Online Appendix B).

The mean age of patients in the outpatient cohort was 67.7 years, slightly younger than the 70.8 years in the inpatient cohort. Furthermore, just over half of the outpatient cohort was male (50.4%), compared to only 41.2% in the inpatient cohort. Diabetes was more common in the inpatient cohort (20.7%) than that in the outpatient cohort (17.2%). Additionally, the proportion of patients with an American Society of Anaesthesiologists (ASA) score of three or greater was 44.9% in the outpatient cohort and 55.1% in the inpatient cohort. The mean BMI was similar in both cohorts, at 30.1 kg/m^2^ for the outpatient group and 30.6 kg/m^2^ for the inpatient group. Lastly, current smokers were more prevalent in the outpatient cohort (6.9%) than in the inpatient cohort (5.4%).

### Post-operative complications

**Figure 2.**
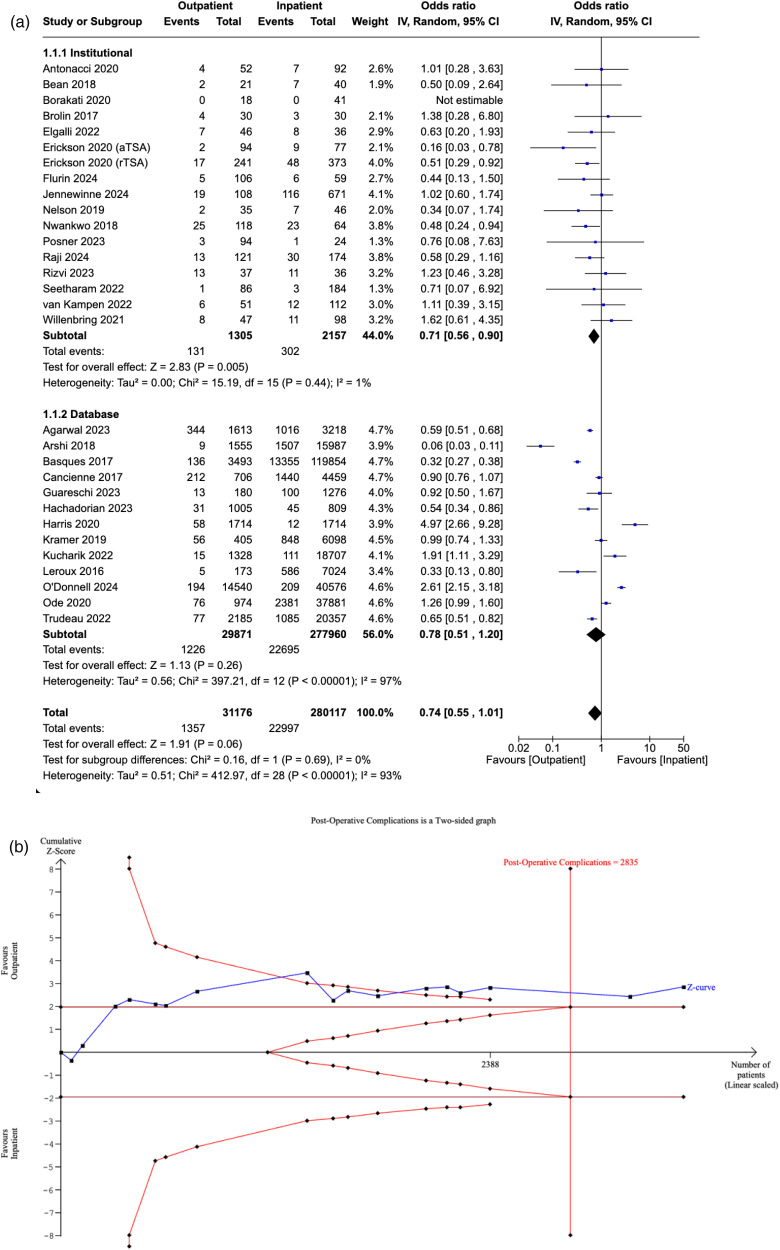
(a) Forest plot comparing post-operative complications between outpatient and inpatient shoulder arthroplasty. (b) Plot comparing post-operative complications between outpatient and inpatient shoulder arthroplasty.

Thirty studies reported the incidence of post-operative complications in both the outpatient and inpatient cohorts. No significant difference in post-operative complications was detected between patients who underwent outpatient and inpatient SA (OR: 0.74, [95% CI: 0.55–1.01], P = 0.06). A post-hoc sensitivity analysis examining post-operative complications specifically in studies reporting same-day discharge also found no significant difference between the two cohorts (OR: 0.76 [95% CI: 0.56–1.05], P = 0.09). Our subgroup analysis revealed a significant difference amongst institutional studies (OR: 0.71, [95% CI: 0.56–0.90], P = 0.005), which was not sustained in database studies (OR: 0.78, [95% CI: 0.51–1.20], P = 0.26). Furthermore, no significant differences in the OR were found between the two subgroups (P = 0.69) (Figure 2A).

The RIS was estimated to be 2835 patients. The Z-curve crossed the conventional boundaries, reinforcing the significant relationship favouring the outpatient cohort, as demonstrated by our meta-analysis. The curve also crossed the conventional boundaries, suggesting that the evidence from our meta-analysis is sufficient to reject the null hypothesis, with minimal risk of Type I error (Figure 2B).

### Surgical complications

Surgical complications arise directly from the procedure or implanted prosthesis. Examples include periprosthetic fractures, nerve palsy, aseptic loosening, dislocation, haematoma, and surgical site infections.^52^

**Figure 3.**
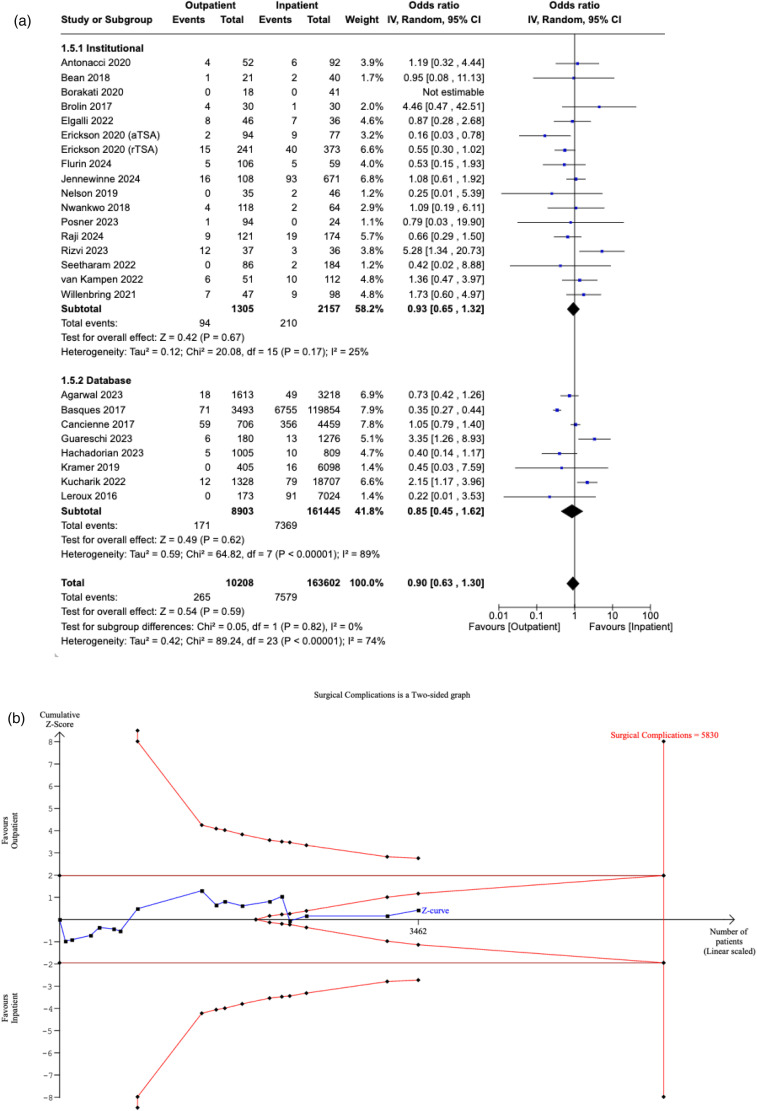
(a) Forest plot comparing surgical complications between outpatient and inpatient shoulder arthroplasty. (b) Plot illustrating results for trial sequential analysis (TSA) of surgical complications in institutional studies.

Twenty-five studies reported surgical complications in the outpatient and inpatient cohorts. No significant difference in surgical complications was observed between the two cohorts (OR: 0.90, [95% CI: 0.63–1.30], P = 0.59), even after post-hoc sensitivity analysis examining studies reporting same-day discharge (OR: 1.00, [95% CI: 0.74–1.34], P = 0.62). Subgroup analysis reinforced similar findings of non-significance in studies utilising institutional data (OR: 0.93, [95% CI: 0.65–1.32], P = 0.67) and databases (OR: 0.85, [95% CI: 0.45–1.62], P = 0.62), respectively. No significant difference was observed between the two subgroups (P = 0.59) (Figure 3A).

The RIS was estimated to be 5830 patients. The Z-curve did not cross the conventional boundaries, indicating a non-significant difference in surgical complications between outpatients and inpatients. However, it did cross the futility boundaries, suggesting that further studies are unlikely to yield a significant effect. Since the curve did not cross the alpha-spending boundaries, the meta-analysis is still deemed non-conclusive. Until the Z-curve intersects the alpha-spending boundaries, the risk of Type II error remains (Figure 3B).

### Medical complications

Medical complications refer to systemic issues that arise post-operatively and are not directly related to the surgery itself. Examples include deep vein thrombosis, acute kidney injury, angina, atrial fibrillation, stroke, and sepsis.

**Figure 4.**
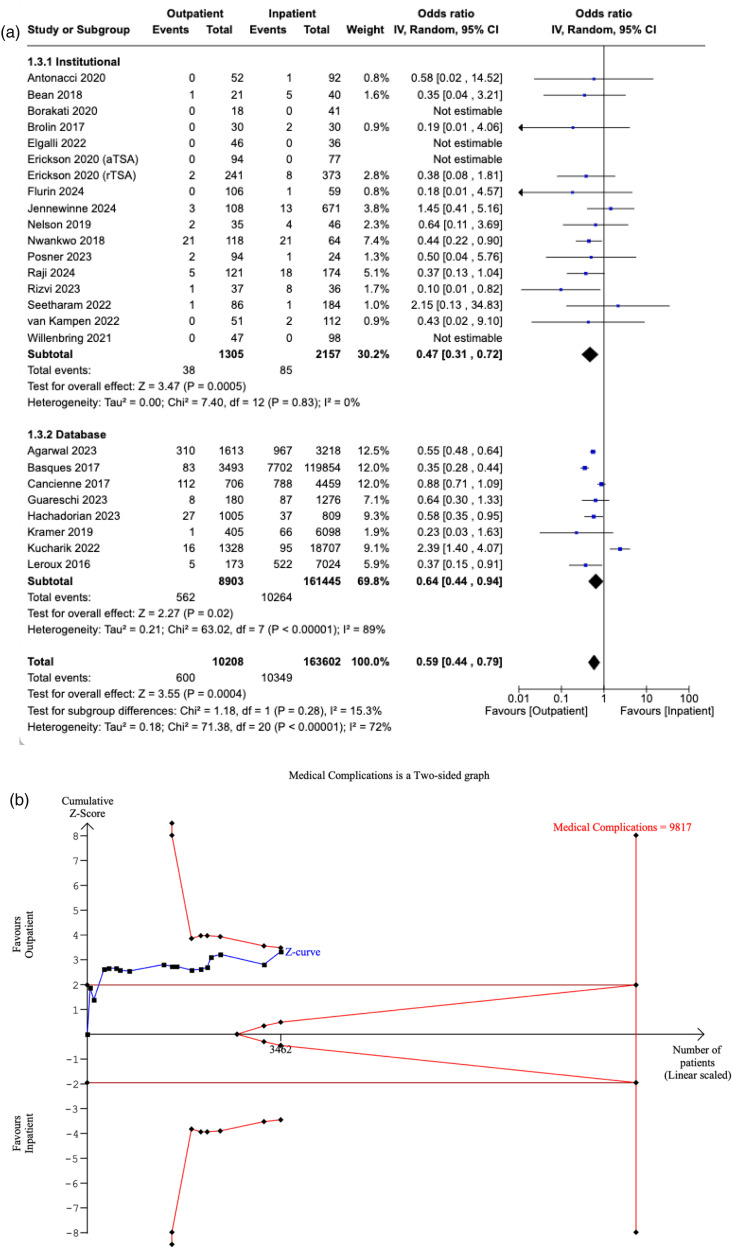
(a) Forest plot comparing medical complications between outpatient and inpatient shoulder arthroplasty. (b) Plot illustrating results for trial sequential analysis (TSA) of medical complications in institutional studies.

Twenty-five studies reported medical complications in both cohorts. A statistically significant decrease in medical complications was noted in outpatient SA compared to the inpatient setting (OR: 0.59, [95% CI: 0.44–0.79], P = 0.0004). Sensitivity analysis of studies reporting same-day discharge revealed an OR of 0.64 ([95% CI: 0.48–0.87], P = 0.004). Furthermore, the subgroup analysis identified that a significant decrease in medical complications in the outpatient setting was sustained in institutional studies (OR: 0.47, [95% CI: 0.31–0.72], P = 0.0005) and database studies (OR: 0.64, [95% CI: 0.44–0.94], P = 0.02). There was no significant difference between the subgroups (P = 0.28) (Figure 4A).

The RIS was estimated to be 9817 patients. The Z-curve crossed the conventional boundaries, suggesting a statistically significant difference favouring the outpatient cohort. However, the curve did not cross the alpha-spending boundaries, meaning our meta-analysis is inconclusive and carries the risk of a Type I error (Figure 4B).

### Readmission

**Figure 5.**
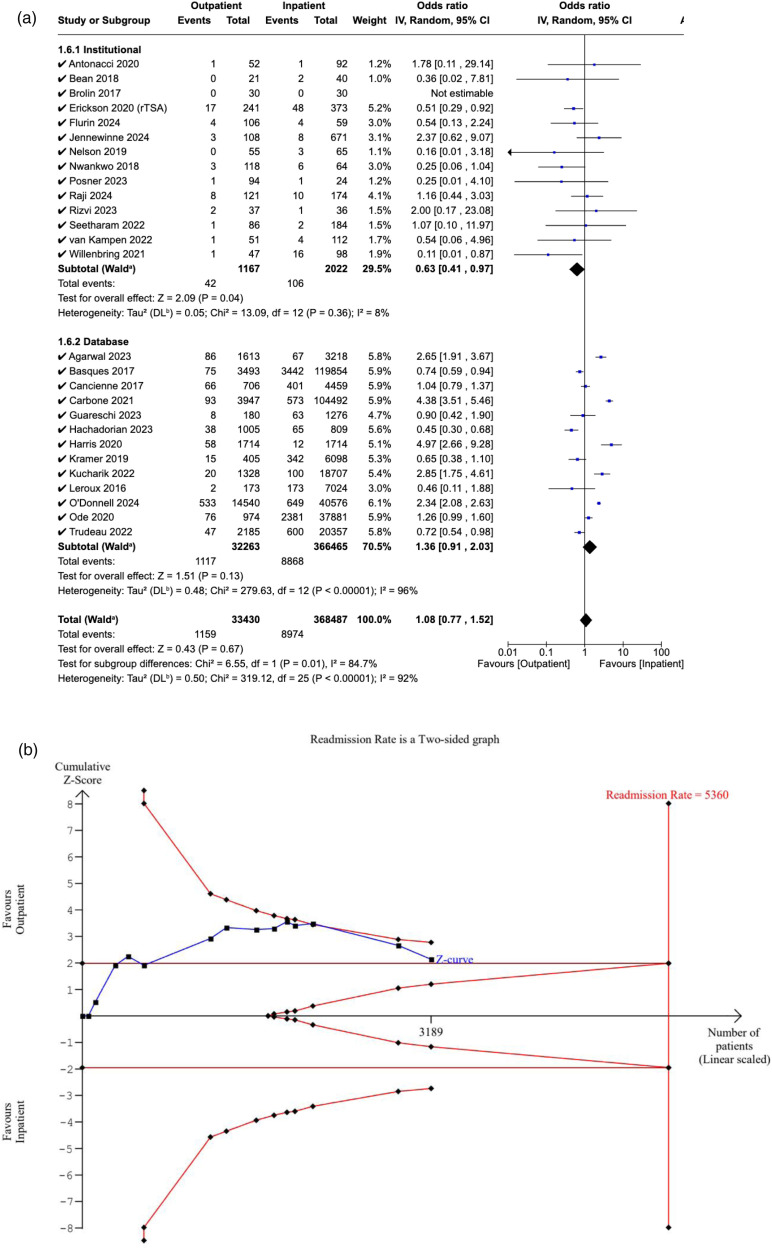
(a) Forest plot comparing readmission between outpatient and inpatient shoulder arthroplasty. (b) Plot illustrating results for trial sequential analysis (TSA) of readmission in institutional studies.

Twenty-seven studies reported the 90-day readmission rates of the outpatient and inpatient cohorts. No significant difference was detected between the two cohorts (OR: 1.08, [95% CI: 0.77–1.52], P = 0.67). Similarly, non-significance was also observed in studies specifically reporting same-day discharge (OR: 1.10, [95% CI: 0.75–1.61], P = 0.62). In studies reporting institutional data, readmission rates were significantly lower in the outpatient cohort (OR: 0.63, [95% CI: 0.41–0.97], P = 0.04). In comparison, the pooled OR of database studies was 1.36 ([95% CI: 0.91–2.03], P = 0.13), which indicates non-significance. A significant difference was detected between the pooled ORs of the two subgroups (P = 0.01) (Figure 5A).

The RIS was estimated to be 5360 patients. The Z-curve crossed the conventional boundaries, which indicates a significant difference in readmission rates favouring the outpatient cohort. However, it did not cross the alpha-spending boundaries, rendering our meta-analysis inconclusive, with a risk of Type I error (Figure 5B).

### Revision surgery

**Figure 6.**
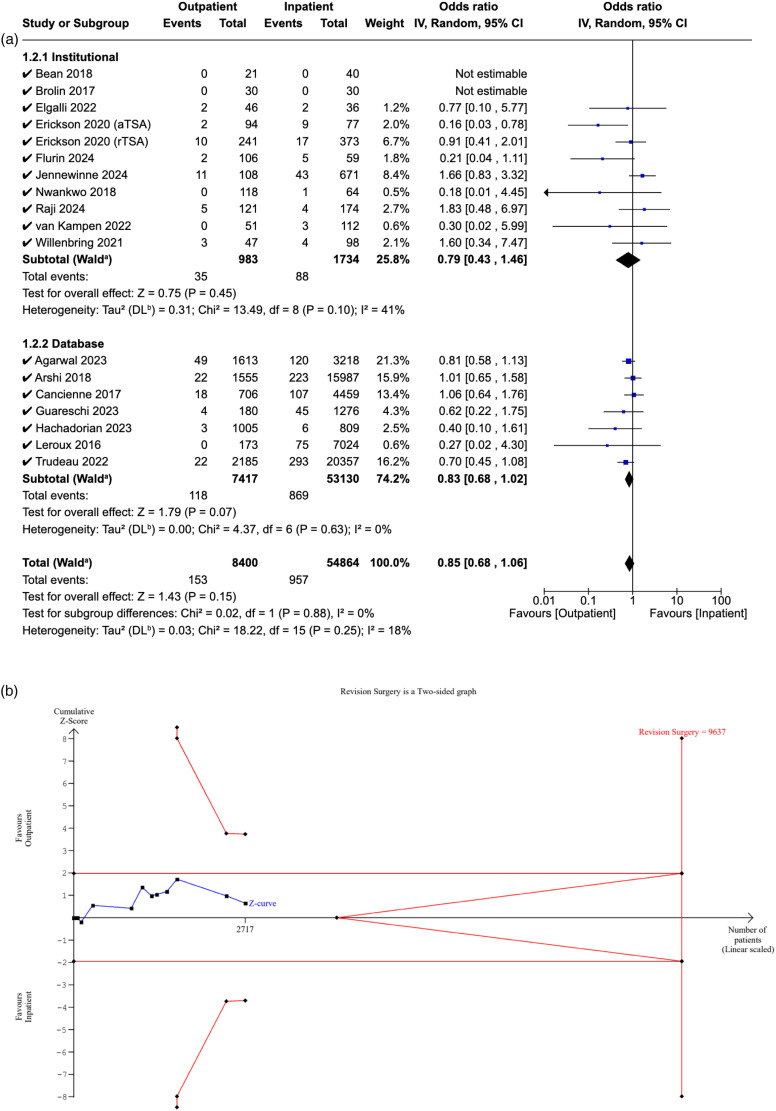
(a) Forest plot comparing revision surgery between outpatient and inpatient shoulder arthroplasty. (b) Plot illustrating results for trial sequential analysis (TSA) of revision surgery in institutional studies.

Eighteen studies reported the 90-day revision rates in both cohorts. The pooled OR for all studies was 0.85 ([95% CI: 0.68–1.06], P = 0.15), suggesting no significant difference in rates between the two cohorts. When analysed separately, no significant difference in revision rates was noted in institutional (OR: 0.79, [95% CI: 0.43–1.46], P = 0.45), or database studies (OR: 0.83, [95% CI: 0.68–1.02], P = 0.07), or between the two subgroups (P = 0.88) (Figure 6A).

The RIS was estimated to be 9637 patients. The Z-curve did not cross the conventional boundaries, demonstrating non-significance in revision rates between the outpatient and inpatient cohorts. Additionally, it did not cross the alpha-spending boundaries, suggesting that the evidence from the meta-analysis is inconclusive, with a risk of Type II error (Figure 6B).

### Emergency department attendance

**Figure 7.**
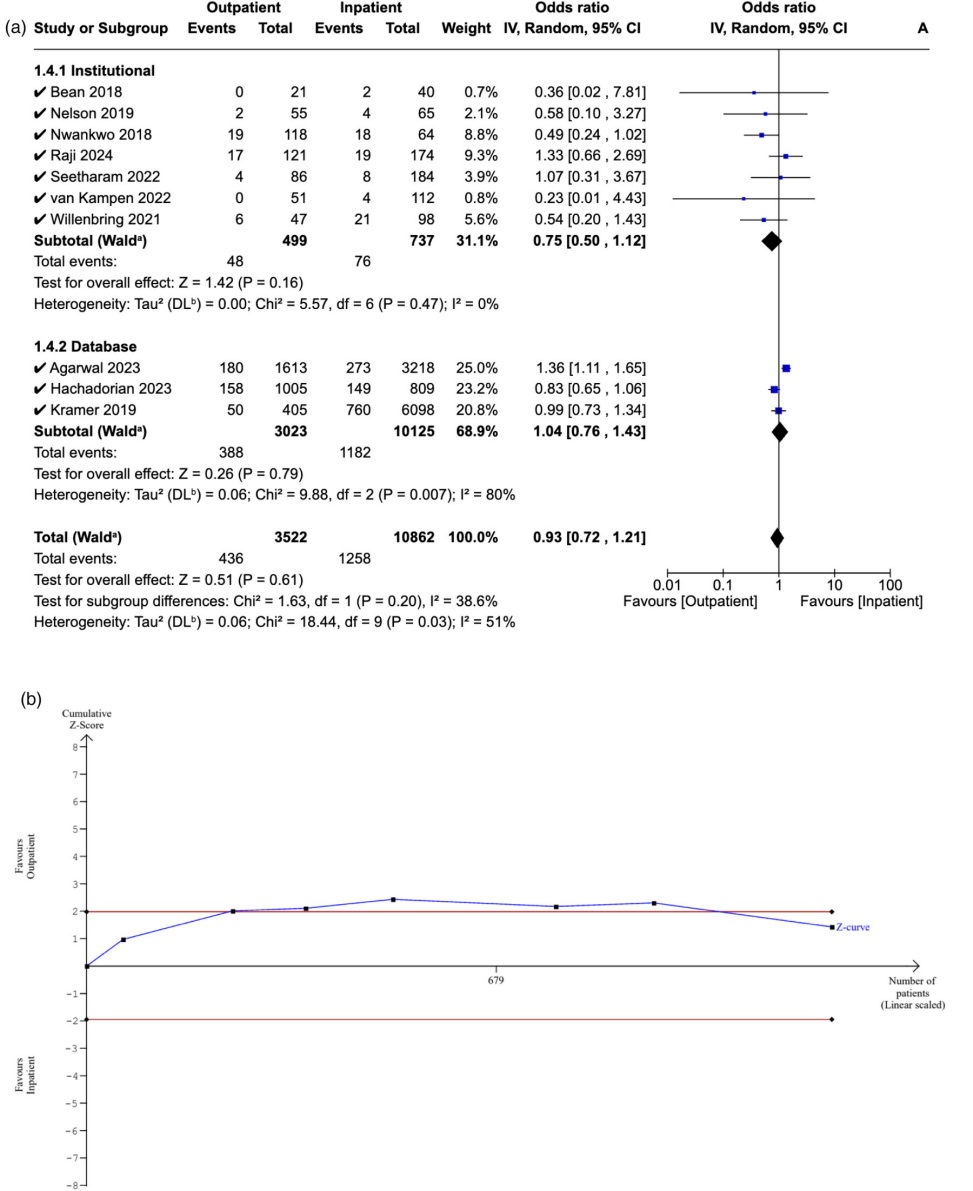
(a) Forest plot comparing emergency department between outpatient and inpatient shoulder arthroplasty. (b) Plot comparing emergency department between outpatient and inpatient shoulder arthroplasty.

Ten studies reported 90-day ED attendance in both cohorts. There was no significant difference in attendance rates between the outpatient and inpatient groups (OR: 0.93, [95% CI: 0.72–1.21], P = 0.61), which persisted in the post-hoc sensitivity analysis (OR: 0.94, [95% CI: 0.72–1.22], P = 0.64). Additionally, no significant difference was observed in institutional studies (OR: 0.75, [95% CI: 0.50–1.12], P = 0.16), database studies (OR: 1.04, [95% CI: 0.76–1.43], P = 0.68), or between them (P = 0.61) (Figure 7A).

The RIS could not be estimated due to the limited sample size. The Z-curve initially crossed the conventional boundaries but later returned, indicating a non-significant difference in ED attendance rates between the outpatient and inpatient cohorts. However, the inability to generate alpha-spending boundaries implies that our meta-analysis does not meet the RIS and is therefore deemed inconclusive and at risk of Type II error (Figure 7B).

### Procedural costs

Eight studies^22,26,28–30,34,46^ compared various cost components in outpatient and inpatient SA (Table 2). Ode et al.^44^ reported that the median overall cost for outpatient cases ($37,395; IQR: $41,327-$87,881) was significantly lower than that for inpatient cases ($62,905; IQR: $41,327-$87,881, P < 0.0001). Interestingly, when outpatient cases were further divided into hospital-based outpatient departments and ambulatory centres, a significant difference in median costs was observed, with the ambulatory setting yielding lower costs ($31,790 vs $55,990, P < 0.0001).^44^ Even after excluding inpatient-specific charges, Gregory et al. found a significant 41.1% decrease in overall costs in outpatient SA compared with the inpatient setting. Regarding itemised reimbursements,^34^ Cancienne et al. found that outpatient SA was associated with significantly lower laboratory costs, physical therapy and occupational therapy costs, and narcotic prescription costs.^28^ However, the antiemetic and anticoagulation prescription costs were considerably higher in the outpatient cohort. Additionally, despite the cost advantages of outpatient SA, Agarwal et al. found that marginal savings compared to inpatient SA progressively decreased at the 30-day, 90-day, and 1-year post-operative intervals.^22^

**Table 2.** Differences in procedural costs between outpatient and inpatient shoulder arthroplasty.

Author	Overall Cost	Other Key Findings
Agarwal et al., 2023, USA^22^	**Outpatient**: $16,941.34 **Inpatient**: $20,583.80 (P < 0.001)	**•** The marginal cost savings between outpatient and inpatient SA decreased over time: 46.5% at 30 days post-operatively, 37.8% at 90 days, and 17.7% at 1 year.
Borakati et al., 2023, United Kingdom^26^	**•** Median savings of outpatient arthroplasty were £529 (IQR: 247.33 – 789.00, P < 0.0001)	
Cancienne et al., 2017, USA^28^	**Outpatient**: $14,722 ± $2806 **Inpatient**: $18,336 ± $3082 (P < 0.0001)	**•** Outpatient SA was associated with significantly reduced PACU costs (P < 0.0001), laboratory costs (P < 0.0001), physical therapy and occupational therapy costs (P < 0.0001), and narcotic prescription costs (P < 0.0001) than inpatient SA. **•** Prescription costs for antiemetics (P = 0.001) and anticoagulants (P = 0.014) were greater in outpatient SA.
Carbone et al., 2021, USA^30^	**Outpatient**: $16,130 ± $6835 **Inpatient**: $12,894 ± $6178 (P < 0.001)	
Cronin et al., 2022, USA^29^	**Outpatient**: $8832 **Inpatient**: $8841 (P = 0.97)	**•** Non-implant total costs were significantly lower for outpatient ($2624) versus inpatient SA ($3,369, P < 0.0001)
Gregory et al., 2019, USA^34^	**Outpatient**: $22,907 ± $13,599 **Inpatient**: $76,109 ± $48,981 (P < 0.001)	**•** Costs for both inpatient and outpatient SA have been increasing from 2015 – 2015 **•** The overall cost of outpatient SA remains 41.1% lower than inpatient SA after excluding patient-specific charges
Ode et al., 2020, USA^44^	**Outpatient**: $37,395 (IQR: $21,976-$61,775) **Inpatient**: $62,905 (IQR: $41,327 – $87,881, P < 0.001)	**•** Outpatient SA performed in ambulatory centres ($31,790, IQR: $19,842 – $59,916) incurred significantly lower costs than those performed in hospital-based outpatient settings ($55,990, IQR: $39,446 – $63,043, P < 0.001)
Raji et al., 2024, USA^46^	**Outpatient**: $69,892.85 ± $10,939.04 **Inpatient**: $70,270.35 ± $15,358.18 (P = 0.82)	**•** No significant difference in costs were found between outpatient and inpatient SA when patients across three age groups: < 65 years (P = 0.69), 65 – 75 years (P = 0.63), > 75 years (P = 0.42).

## Discussion

This systematic review and meta-analysis compared various clinical outcomes and procedural costs between outpatient and inpatient SA. Several reviews^12–14^ have already been conducted on the topic in the past but their last search date was limited to 2020. Our review serves as an update to the previous literature, incorporating almost 20 additional studies compared to Perera et al.^12^ and Cimino et al.^14^ In addition, to the best of our knowledge, this study represents the inaugural application of trial sequential analysis in the context of outpatient SA, to assess whether the existing evidence is sufficient or if further evidence is required. Overall, this systematic review demonstrated that outpatient SA is associated with lower rates of medical complications, non-inferior rates of total post-operative complications, surgical complications, readmission, revision surgery, ED attendance, and significantly lower procedural costs compared to the traditional inpatient approach.

Regarding the safety of outpatient SA, our findings are largely in agreement with the previous review by Perera et al.^12^ The exceptions were total post-operative complications, which Perera et al.^12^ reported as significantly decreased in the outpatient cohort, and medical complications, for which no significant difference was detected between the two groups. Our subgroup analysis also revealed no significant difference between institutional and database studies for most clinical outcomes, with the exception of readmission rates, which were lower in institutional studies. While these results are promising, it is important to recognise that they are accompanied by substantial selection bias within the included studies, as patients in the outpatient cohort were, on average, younger, more likely to be male, less likely to have diabetes or high ASA classifications, and had a lower BMI. Several authors^22,27,28,37^ matched inpatient and outpatient cohorts to mitigate this bias, all demonstrating comparable safety outcomes between the two groups. Nonetheless, outpatient SA should not be viewed as a replacement for the traditional inpatient model, but rather as a means to combat the rising pressures and challenges faced by healthcare institutions.^53,54^ Particularly in the UK, bed shortages are a critical issue, with approximately 19 in 20 beds in adult wards occupied at all times, many of whom are already medically fit for discharge.^55,56^ Coupled with the ongoing staffing shortfalls within the National Health Service (NHS), this places major pressure on hospitals across the UK, culminating in inefficient, untimely, and costlier patient care.^57^ In this context, outpatient SA offers a feasible solution that eases the burdens placed on healthcare institutions such as the NHS, while maintaining safe and optimal outcomes for patients. However, the selection bias highlighted within the outpatient cohort emphasises the importance of careful patient selection to achieve such outcomes.

Selecting patients suitable for outpatient shoulder arthroplasty is a judgement primarily made by the surgeon, utilising a range of patient factors, including age, comorbidities, social support, and patient preference to make an overall assessment.^58^ However, there are currently no established patient selection criteria for surgeons to follow, leading to variability in decision-making and, in some cases, misjudgements that may inadvertently jeopardise patient safety. With only 20.7% of shoulder surgeons performing outpatient SA, Brolin et al. reported that surgeons’ concerns regarding medical complications and readmission were amongst the biggest barriers towards its successful implementation.^59^ The burden placed on surgeons to accurately select patients solely based on their clinical judgement serves as an additional deterrent. With this in mind, identifying the exact patient factors that heighten the risk of adverse events in outpatient SA is crucial to assist surgeons in making more informed risk stratification decisions. Willenbring et al. found that age was the strongest predictor of surgical complications, with a 14% annual increase in risk.^49^ In another study, Cancienne et al. highlighted diabetes mellitus, peripheral vascular disease, congestive heart failure, chronic lung disease, depression, and chronic anaemia as clinically-significant risk factors for 90-day readmission in patients undergoing outpatient SA.^28^ Jennewine et al. expanded on such findings and developed patient selection algorithms to further optimise patient safety in outpatient SA, which demonstrated major promise in maintaining low rates of peri-operative complications and readmission.^38^ Similarly, Fournier et al. reported low complication rates and no readmissions among 61 patients undergoing outpatient SA by developing a patient selection algorithm that primarily focused on age and cardiopulmonary comorbidities.^60^ An important factor to consider when integrating published patient selection algorithms into routine clinical practice is what their definition of *outpatient* SA actually is.^38,60^ Our inclusion criteria defined *outpatient* status as a length of stay less than 24 h; however, across the included studies, its definition varied between this and same-day discharge. Same-day discharge is likely to have more restrictive selection criteria to account for the concerns and risks associated with earlier discharge. As a result, these algorithms may not be entirely generalisable across institutions and should therefore be used with caution. Moreover, risk prediction tools, such as those proposed by Goltz et al.^61^ and Khan et al.,^62^ have shown promise in assisting clinicians identify suitable candidates for outpatient shoulder arthroplasty. However, these models have been tested on limited sample sizes and require validation on a larger, more diverse population to assess its feasibility.

Beyond safety, our systematic review highlighted another key finding: outpatient SA is more cost-effective than inpatient SA. Six studies documented lower overall procedural costs in outpatient SA compared to the inpatient setting.^22,26,28,30,34,44^ Conversely, Raji et al.^46^ and Cronin et al.^29^ found that the overall hospital charges were comparable between the two cohorts. Nonetheless, given the aforementioned challenges faced by healthcare institutions, these cost savings are especially. A systematic review by Crawford et al. highlighted operating room, inpatient admission, and floor charges as the main drivers behind cost reductions in outpatient surgical procedures.^63^ Additionally, inpatient SA procedures are often more complex, involving multi-morbid patients who require intensive perioperative monitoring and medical management, further contributing to the stark difference in costs between the two approaches. Moving on, Borakati et al.^26^ was the only study to report procedural costs in the United Kingdom, documenting median savings of £529, which drastically differed from figures reported in the USA, despite being statistically significant. This is partly because the only cost variables recorded were catheter insertion and removal, analgesic costs, and inpatient admission.^26^ Nonetheless, lower inpatient admission costs in the UK compared to the USA are likely a leading factor behind these disparities.^64^

For our trial sequential analysis, we decided to only include institutional studies, as overlapping sources in database studies could hinder the accuracy of the findings. Only our meta-analysis of total post-operative complications met the required information size and was therefore regarded as conclusive, with the remaining deemed inconclusive and at risk of either Type I or Type II error. Despite multiple newly published studies, these findings underscore the crucial need for further research to build on existing findings so that, eventually, they can be accurately relied on by surgeons in clinical practice. Future research should ideally focus on conducting multi-centre, prospective, randomised controlled trials, with longer follow-ups and larger sample sizes to strengthen the current evidence base.

### Limitations

Our study had several limitations. First, almost all studies were retrospective, classified as either Level III or IV evidence, and carried a *serious* risk of bias, primarily due to patient selection and confounding factors. Ultimately, this hinders the validity and generalisability of our findings, particularly the meta-analyses and corresponding trial sequential analyses. Another major limitation was the considerable overlap in the databases used amongst the included studies. Multiple studies used the ACS-NSQIP database, and although their data collection timelines varied, there remained patient overlap between these studies. For this reason, a pre-planned subgroup analysis was conducted to compare studies using databases and institutional records, and database records were excluded from the trial sequential analysis to ensure the reliability of the findings. Furthermore, compared to other outcomes, there were substantially fewer studies that reported ED attendance. As a result, the statistical power was too low to determine the sample size required to confidently accept the findings. This is a valuable outcome to evaluate as it can provide indirect insights into patient behaviours following outpatient surgeries. Outpatient surgeries are associated with heightened levels of pre-operative anxiety and stress, and this may manifest in increased ED attendance.^65,66^

## Conclusion

This systematic review and meta-analysis demonstrated that, with careful patient selection, outpatient shoulder arthroplasty is cost-effective and can yield comparable outcomes to the inpatient approach. Nonetheless, the trial sequential analyses indicated that our meta-analyses are not yet conclusive, underscoring the need for further research, preferably in the form of RCTs to strengthen the existing evidence base.

## Supplemental Material

sj-docx-1-sel-10.1177_17585732251349754 - Supplemental material for Outpatient shoulder arthroplasty: An updated systematic review, meta-analysis, and trial sequential analysis on clinical outcomes and cost-effectiveness Supplemental material, sj-docx-1-sel-10.1177_17585732251349754 for Outpatient shoulder arthroplasty: An updated systematic review, meta-analysis, and trial sequential analysis on clinical outcomes and cost-effectiveness by Ahmed Al-Saadawi, Sam Tehranchi, Richard Benson, David Rose and Obinna Jude Nzeako in Shoulder & Elbow
