# Supplementary material for: Outpatient shoulder arthroplasty: An updated systematic review, meta-analysis, and trial sequential analysis on clinical outcomes and cost-effectiveness
Source: Shoulder Elbow. 2025 Jun 25;18(2):203–24. doi: 10.1177/17585732251349754 (PMC12202397; doi:10.1177/17585732251349754)
Supplement: sj-docx-1-sel-10.1177_17585732251349754 - Supplemental material for Outpatient shoulder arthroplasty: An updated systematic review, meta-analysis, and trial sequential analysis on clinical outcomes and cost-effectiveness [file sj-docx-1-sel-10.1177_17585732251349754.docx]

**Search Strategy:**

1. exp/ Arthroplasty, Replacement, Shoulder

2. shoulder arthroplast* :ti,ab.

3. hemiarthroplast* :ti,ab.

4. exp/ Outpatients

5. exp/ Ambulatory Surgical Procedures

6. outpatient* :ti,ab.

7. same day* :ti,ab.

8. day care* :ti,ab.

9. day surgery* :ti,ab.

10. early discharge* :ti,ab.

11. 1 **OR** 2 **OR** 3

12. 4 **OR** 5 **OR** 6 **OR** 7 **OR** 8 **OR** 9 **OR** 10

13. 11 **AND** 12
